# Supplementary material for: Are carbon emissions trading and green financial instruments synergistic? -Comprehensive quantitative research based on content analysis
Source: PLoS One. 2024 Mar 7;19(3):e0298601. doi: 10.1371/journal.pone.0298601 (PMC10919601; doi:10.1371/journal.pone.0298601)
Supplement: S1 Data — (ZIP) [file pone.0298601.s001.zip › Data/地级市Z_F工作报告.zip!/╜¡╬≈/╛░╡┬╒≥╩╨/└┤╫╘╣1⁄2╓┌║┼í╛┬φ┐╦╩2╛▌═°í┐_2016.docx]

各位代表：

　　现在，我代表市人民政府向大会作工作报告，请予审议，并请各位政协委员和其他列席人员提出意见。

　　一、“十二五”时期经济社会发展主要情况

　　“十二五”期间，我市经受了经济下行压力加大的严峻考验，经济社会发展取得巨大成就。在省委、省政府和市委的坚强领导下，我们认真贯彻落实省委十六字方针和市委战略部署，主动适应经济发展新常态，稳增长、促改革、调结构、惠民生，圆满完成各项目标任务。预计2015年生产总值800亿元，比“十一五”末增长62.7%；人均生产总值8000美元，增长58.2%；三次产业比例调整到7.1：57.5：35.4；财政总收入111.6亿元，增长109.9%；固定资产投资691亿元，增长101%；社会消费品零售总额264亿元，增长88.1%；人口自然增长率保持在7.6‰以下；节能减排完成计划目标。通过“十二五”时期全市上下的奋力拼搏，特别是近两三年来全力实施工业、招商、城建“三大战役”，我市主要经济指标增幅排位企稳前移，部分指标实现翻番，城市面貌发生可喜变化，项目建设取得重大突破，主导产业在全省格局中地位更加突显，发展基础更加牢固，经济增长爆发力加速形成。

　　（一）转型升级迈出坚实步伐，经济发展量质双升

　　工业经济加速崛起。预计规上工业增加值（含军工）303亿元，比“十一五”末增长86.9%；主营业务收入超亿元企业达到159户，昌飞、焦化突破百亿元。产业集聚水平进一步提升，优势主导产业完成主营业务收入960亿元，比重达到78%。直升机、精细化工和陶瓷产业集群列入省重点产业集群，精细化工产业集群列入省级工业示范产业集群。一批重大产业项目先后建成。北汽重组昌汽并投资140亿元建设南方总部基地，一期工程竣工投产；组建江直投公司并与北京通航签订60 亿元战略重组协议，总装厂房主体工程完工，昌飞直升机总装园厂房主体工程完工；陶瓷股份公司成为中国陶瓷行业最具竞争力十强企业，名坊园一期正式开园；华意压缩新600项目一期竣工投产，成为全球最大家用压缩机制造商；天新药业维生素B1和B6、宏柏化学硅烷偶联剂市场份额全球第一，黑猫炭黑市场份额全球第四、亚洲第一，世龙实业AC发泡剂市场份额全国第一。通过扩园强区和权力下放，园区对工业经济的承载能力大幅提高。高新区直升机产业基地成为国家战略性新兴产业区域集聚发展等“两试点两基地”，陶瓷工业园获批国家新型工业化陶瓷制品产业示范基地，乐平工业园获批国家级精细化工高新技术产业化基地。

　　现代服务业蓬勃发展。深入推进国家服务业综合改革试点，现代服务业对经济的拉动作用日益突出。预计服务业增加值283亿元，比“十一五”末增长56.6%，占生产总值比重提高4.4个百分点。旅游产业发展迅速，接待游客、旅游总收入分别年均递增18.56%、31.3%，达到3100万人次和260亿元。新增3个4A景区，古窑成功创建5A景区。连续3年获得全省旅游商品博览会特等奖。金融改革发展不断加快，金融业增加值18.56亿元、增长2.1倍。引进了3家银行、3家保险公司和2家证券公司设立分支机构，在全国首批设立2家村镇银行。世龙实业、富祥药业首发上市。文化产业生机勃发，老城保护区列入省级服务业集聚区。陶溪川国际陶瓷文化产业园核心区基本完工，部分场馆对外开放。连续12届成功举办的瓷博会，成为深化贸易合作、扩大人文交流、展示瓷都形象的重要会展品牌。电子商务爆发式增长，跻身全国电商百佳城市，网商指数全省第一，美淘电商孵化基地、唐龙陶瓷成为全省电商示范基地（企业）。

　　农业经济稳中有进。预计农业总产值88亿元，较“十一五”末增长22%。粮食总产63.6万吨，增长10.6%，实现“十二连丰”。蔬菜播种面积、产量分别增长9.5%、19.5%。茶叶种植面积、总产分别增长28.4%、42.6%。浮梁茶品牌知名度进一步提升，入选全省重点整合的“四绿一红”茶叶品牌。市级以上龙头企业119家，增加70%，带动33万户农民增收。获得2个国家农产品地理标志，龙头企业拥有省级以上品牌34个。乐平现代农业示范区晋升为国家级。完成病险水库除险加固401座，新增高标准农田37.25万亩。

　　（二）城乡面貌发生巨大变化，生态家园初具雏形

　　中心城加速扩容提质。建成区面积达到86平方公里，比“十一五”末增加19.4%。规划体系日趋完善。完成新一轮城市总体规划修编及相关专项规划编制，控制性详规覆盖率超过80%。古镇风貌初步展现，编制完成《历史文化名城保护规划》，并按规划要求对老街区、老厂区和老窑址等实施了“点、线、面”立体控制和保护。御窑景巷完成河堤景观区及沿街商业用房建设。明清窑作陶瓷文化遗址及彭家弄、三闾庙等历史文化街区保护项目主体完工，改造维修了一批主干道及里弄、桥梁、排水系统，新建了一批街心花园和停车场。城市新框架全面拉开。昌南拓展区南片区主骨架基本成型，主要公建项目进展顺利，绿地陶瓷文化旅游城全面开工。高铁商务区开工建设。赣东北物流园进展顺利，国际会展中心、国大汽车城、罗家滩收费站西迁等项目相继完工。规划了昌江、珠山两个区域副中心并启动前期工作。城市管理水平明显提升。全面启动“五城同创”并以此为抓手整顿市容市貌，开展了规范陶瓷市场、治理交通拥堵、整顿城市建设秩序等16个方面综合整治活动，重拳打击“两违”。完成城市管理体制调整，推行政府服务外包，建成数字化城市管理平台并投入使用，初步形成“市级监管、区级实施、街道和社区为重要参与主体”的城市管理新格局。以珠山中路、珠山西路、莲社北路为核心打造文明创建示范区，蝉联全国文明城市提名城市。

　　城乡一体化协调推进。户籍制度改革稳步实施，农业转移人口市民化进程加快，城镇化率64%，提高7.69个百分点。累计投入资金2.3亿元用于新农村建设，惠及32万农村人口。7个镇村联动试点镇建设初见成效。在5个省级百强中心镇开展了扩权强镇试点。与省水投集团合作的城乡一体化供水项目加快推进。县乡公路新建和改造力度加大，行政村公路通达率100%。农村生活垃圾专项治理效果显著，村庄周边和干道沿线卫生环境极大改善；2个中心镇污水处理设施及污水管网建设完工；乐平市、浮梁县、昌江区获评全省新农村建设、清洁工程等先进单位。

　　基础设施建设取得重大突破。一批事关景德镇长远发展的重大项目陆续开工或竣工。浯溪口水利枢纽工程进展顺利，主体工程混凝土浇筑量完成96%。九景衢铁路全面开工，已进入铺轨阶段。西瓜洲段防洪堤全面建成，吕蒙堤主体工程基本完工，瓷都大桥至观音阁段及西河防洪堤陆续开工。飞虹大桥及飞虹大道开工建设。206国道完成大中修。洋湖水厂搬迁改造项目开工。景德镇机场搬迁和电厂2台100万千瓦机组扩建工程已开展前期工作。昌景黄高铁初步列入国家“十三五”路网规划。共争取到全国低碳试点城市、全国老工业城市调整改造以及资源型城市转型延续5年等政策，获得中央各类资金支持70亿元。

　　生态文明建设成效显著。单位生产总值能耗累计下降16%，主要污染物排放总量完成削减任务。2015年地表水断面达标率97.4%、全年空气质量优良天数329天。宋家山垃圾填埋场扩容有序推进，垃圾焚烧发电厂完成主体结构。城市供水水源工程启动实施。第二污水处理厂完成扩容，污水管网一期工程完工。累计新增造林34.4万亩，森林覆盖率提高到65.73%，建成区绿化覆盖率由42%提高到52.1%。建成国家级森林公园2个、省级森林公园5个。水生态文明试点建设和自主创建加快推进，一批乡镇、村成为省级试点。浮梁县成功创建国家级生态县，并列入全省第一批生态文明先行示范县。高新区成为省级生态工业园区。陶瓷工业园、乐平工业园列入省级生态工业园试点。

　　（三）开放创新向纵深推进，发展活力加速迸发

　　开放型经济生机勃勃。全力打好招商引资大会战，加快“走出去”、“引进来”步伐，通过组建专业队伍、完善工作机制，营造了浓厚氛围，激活了招商局面。在积极参加全省重大招商活动的同时，赴发达省市推介产业、对接项目，并在上海、厦门等地成立商会以商招商。五年累计引进内资实现翻番；引进外资6.89亿美元，增长51.76%。落实鼓励出口政策、组织企业参加境内外展会并加快通关一体化，帮助企业扩大外贸出口。五年累计出口49.5亿美元，增长193.6%，其中生产型出口超过77%。

　　对外交流合作持续扩大。荣膺“世界手工艺与民间艺术之都”，加入全球创意城市网络，并借此平台与国内外69个城市建立了广泛联系。成为联合国海陆丝绸之路城市联盟首批创始成员。与国外20个省市建立或深化友好关系，60多个国家或国际组织派员来我市访问。与上饶、鹰潭建立了赣东北扩大开放合作关系。承办了首届驻华外交官“中国文化之旅”交流活动和第六届世界缅华同侨联谊大会，举办了御窑遗址与故宫瓷器对比展、“中国文化遗产美术展”等展览，先后赴10多个国家及国内多个省市开展文化交流活动。

　　深化改革成果斐然。完成部分行政区划调整和新一轮政府机构改革。行政审批制度改革顺利推进，“三单一网”已出台权力清单、责任清单，建成政务服务网并投入运行。整合市场监督管理职能，商事制度完成“三证合一”改革。警务机制改革强力推进。完善了国库集中支付制度，建成社会综合治税信息平台，建立了房地产税费一体化征管和艺术陶瓷税收征管机制，“营改增”试点稳步推进。基本建立了统一规范的公共资源交易平台。数字化综合办公平台投入使用。第一轮行政及参公单位公务用车改革进展顺利。混合所有制经济改革取得突破，开门子肥业首开先河引进民营资本进入上市辅导期。公交公司改制基本完成。出租车行业改革有序推进，基本完成出租车更新换代。市农信社在全省率先改革成立农商银行。完成了国有林场和农垦企业改革。基本完成农村土地确权工作。

　　（四）公共服务能力显著增强，幸福指数持续提高

　　人民生活继续改善。五年民生支出占一般公共预算支出比重56%。城乡居民人均可支配收入分别由16846元、6748元增加到29288元、12817元，年均增长11.7%和13.7%，超过GDP增速。新增城镇就业17.62万人，城镇就业率96%，转移农村劳动力13.78万人。连续11年提高企业退休人员养老金，城乡居民社会养老保险参保率95.9%。城乡低保标准提高到470元、255元。基本医疗保险和新农合参保率分别达到98%、99.5%。完成44681套（户）保障房建设及棚户区改造，超额完成任务，发放廉租补贴9789万元。加大扶贫开发力度，贫困人口累计减少1.46万人。中心城新增公交车100辆、公交线路16条。

　　社会治理不断加强。基层建设水平进一步提升，珠山区获评全国和谐社区建设示范城区，创建了4个全国示范和谐社区和9个全省精品农村社区。积极推进依法信访，信访形势总体稳定。综合性应急救援队伍建设和基层单位应急管理规范化建设进入全省先进行列。大力推进“平安瓷都”建设，立体化治安防控体系逐步完善，各项打击整治行动深入开展，社会治安好转率连续5年全省第一。食品药品安全监管进一步加强。严格落实安全生产责任制，连续14年未发生重特大安全生产事故，打击非法小煤窑连续9年“零开采、零死亡”。

　　各项事业全面进步。义务教育均衡发展，办学条件显著改善。基本普及了学前三年教育。高考上线率、普通高中优质资源率居全省前列。景德镇高专升格为景德镇学院。老年大学新教学楼投入使用。科研经费占GDP比重2%，高于全省平均水平，国家知识产权局批准在我市设立中国陶瓷知识产权维权中心。与中科院上硅所、北京航空大学、浙江大学等国内知名科研院所深化了产学研合作。直升机技术项目入选国家“863”计划。我市列入国家版权示范基地。乐平市被评为全国科普示范县。御窑厂遗址成为国家考古遗址公园并初具规模，启动了大遗址保护和世界文化遗产申报工作。新增5处国家文物保护单位，乐平南窑入选2013年全国十大考古新发现。保护挖掘了寿安金家坞龙窑和湘湖进坑瓷石矿洞遗址，填补和完善了景德镇宋代制瓷业体系。成功承办全国第9个文化遗产日主场城市活动。乐平市被评为全国文化先进县。丽阳镇入选江西十大文化古镇。陶瓷博物馆完成改造并正式开馆。电视人口综合覆盖率99.3%。圆满举办第12届市运会，获得第15届省运会举办权，被评为全国群众体育先进单位。医药卫生体制改革持续深化，在全省率先实现县级公立医院综合改革全覆盖，基本医疗和公共卫生服务能力明显增强。国防动员建设和双拥工作成效明显，实现双拥模范城“五连冠”。此外，供销、气象、水文、减灾、民宗、慈善、人防、档案、统计、地方志、邮政、通讯、烟草等其他各项事业都取得了新的进步。

　　同时，我们深入开展党的群众路线教育实践、“三严三实”专题教育以及“转作风、提效能、促发展”等主题活动，严格落实中央八项规定和国务院“约法三章”，坚决反对“四风”，“三公”经费大幅压减。深入推进依法行政，全面推行政府法律顾问制度，法治政府建设水平迈上新台阶。自觉接受人大依法监督和政协民主监督，认真办理人大代表建议417件、政协提案674件，办理质量不断提高。大力推进简政放权，审批流程不断优化，行政效能持续提升。进一步扩大政务公开，阳光政府建设取得新成效。严格落实廉政责任制，强化了审计监督和行政监察，严肃查处了一批损害群众利益的突出问题。

　　各位代表，以上成绩的取得，是全市人民共同努力的结果，来之不易，值得倍加珍惜。在此，我代表市人民政府，向全市广大干部群众，向各位人大代表、政协委员、各民主党派、工商联、无党派和社会各界人士，向驻景各单位、解放军、武警官兵、公安干警和离退休老同志，向所有关心、支持、参与景德镇建设发展的朋友们，表示衷心的感谢并致以崇高的敬意!

　　在总结成绩的同时，我们也清醒地看到，经济总量较小、综合实力较弱的基本市情没有改变。龙头骨干企业数量较少且有些还处在培育期，产业集聚水平还不够高，新型工业化仍然任重道远。此外，城市建设任务繁重，公共服务供需矛盾突出，社会治理水平亟待提高，部分公职人员存在懒政怠政现象。对此，我们将采取有力措施，认真加以解决。另外，2015年承诺的10件民生实事，由于各种原因还未完全办结，在此我代表市政府向各位代表表示歉意！

　　二、“十三五”时期经济社会发展的指导思想、发展目标和主要任务

　　根据《中共景德镇市委关于制定全市国民经济和社会发展第十三个五年规划的建议》，市政府制定了《景德镇市国民经济和社会发展第十三个五年规划纲要（草案）》，提交本次大会审议。

　　根据《纲要（草案）》，“十三五”时期全市经济社会发展的指导思想是：高举中国特色社会主义伟大旗帜，以马列主义、毛泽东思想、邓小平理论、“三个代表”重要思想和科学发展观为指导，深入贯彻习近平总书记系列重要讲话精神，遵循“四个全面”战略布局，落实省委十六字方针，以“五大发展”理念为引领，以“形、实、魂”为要求，以“提质翻番、率先小康”为目标，复兴千年古镇、重塑世界瓷都、保护生态家园、建设旅游名城，打造一座与世界对话的城市。

　　按照“提质翻番、率先小康”的总目标，综合考虑发展基础、环境和条件，《纲要（草案）》提出，未来五年我市经济社会发展主要目标是：地区生产总值年均增长8.5%以上，到2020年达到1200亿元，人均生产总值突破1.1万美元；财政总收入年均增长10%以上，到2020年达到180亿元；三次产业比例优化调整为5：53：42；科技进步贡献率提高到60%；常住人口城镇化率达到69%，户籍人口城镇化率达到46%；文化产业增加值达到100亿元；森林覆盖率稳定在65%以上，全市主要河流监测断面Ⅰ～Ⅲ类水质提高到85%以上，万元GDP能耗累计下降完成省下达目标任务；城乡居民人均可支配收入年均分别增长9%和10%左右，达到4.5万元和2.1万元；现行标准下贫困人口全面脱贫。

　　围绕上述目标，《纲要（草案）》提出了“十三五”时期的重点任务：

　　第一，强力推进“3＋1”特色优势产业。要加快形成一批极具竞争力的企业，进一步提升陶瓷产业技术创新水平，加速培育陶瓷品牌集群，做强陶瓷产业；扩大整机产能，加快航空零部件和通航产业发展，做大航空产业；扩大整车产能，增强零部件生产实力，提升核心技术竞争力，做优汽车产业。优化旅游产品体系，形成旅游发展新机制，大力发展全域旅游，推动旅游与文化等领域深度融合，做旺旅游产业。继续加大航空、汽车及零部件、旅游文化招商引资力度，举全市之力实施“3＋1”特色优势产业发展战略，到“十三五”末，力争航空、汽车、旅游文化（含陶瓷文化创意）三大产业全口径主营业务收入均突破千亿元。

　　第二，推动现代服务业和传统产业优化升级。要加快发展现代服务业，推动生产性服务业向专业化和价值链高端延伸、生活性服务业向精细化和高品质转变。改造提升传统产业，促进精细化工、生物医药等传统产业转型升级。以数字化、柔性化、智能化为重点，做大家电电子产业集群。坚持用工业化理念发展农业，推动农业特色化、绿色化、生态化和现代化发展。加快推进互联网与经济社会各领域深度融合发展，使“互联网＋”成为创新发展的重要驱动力。

　　第三，培育文化引领新格局。要培育和践行社会主义核心价值观，深化群众性精神文明创建活动，全面提高公民道德素质和社会文明程度。大力发展文化产业，加快推进文化产业提档升级，使文化产业成为支柱产业。正确处理文化遗产传承保护与经济发展、城市建设的关系，延续瓷都历史文脉，留住城市记忆。大力推进基本公共服务体系标准化、均等化建设，加快构建广覆盖、强保障、一体化、可持续的基本公共服务体系。

　　第四，构筑城市发展新支撑。要强化规划的引领作用，树立全域规划的理念，建立覆盖全域、衔接紧密的科学规划体系。坚持拓展区与老城并举、建设与保护并重，着力完善城市功能，提升城市品位。完善交通、信息、水电气、综合管廊等基础设施，提高污水处理和截污能力。加强各种运输方式衔接，畅通对外开放通道，实现与周边城市无缝对接。强化能源总量保障，优化能源结构，构建稳定、安全、经济、清洁的现代能源体系。以“五城同创”为抓手，提高城市管理科学化、规范化、精细化水平。

　　第五，增强创新发展新动力。要增强科技创新能力，优化技术创新体系，全面提升科技创新对经济增长和产业升级的贡献度。强化人才支撑体系建设，加强高端创业创新人才平台建设。放宽政策、放开市场、放活主体，激发各类创新创业主体活力，大力推进大众创业万众创新。全面深化教育领域综合改革，增强教育在创新发展、文化传承中的基础性作用，基本实现教育现代化。

　　第六，持续提升开放水平。要加强对外交流与合作，融入国家开放大战略。强化产业项目承载平台建设，夯实“引进来”工作基础条件，强化“走出去”指导和服务，推进口岸建设，构筑大开放平台。坚持招大引强，积极开展产业对接合作，深入实施对接央企战略，不断拓宽吸纳外资途径，着力提高招商引资的质量和水平。扩大外贸出口规模，提高外贸出口质量，推进对外贸易转型升级。

　　第七，保护美丽生态家园。要建设质量更优的森林生态系统，加大湿地保护和修复力度，增加生态公共服务供给，构建优质自然生态体系。加快推进清洁生产，发展循环经济，倡导绿色消费，构建绿色生产和消费体系。深入实施“净空”、“净水”、“净土”工程，实行最严格的环境保护制度，形成政府、市场、企业、公众多元共治的环境治理体系。严守生态红线，坚持源头严防、过程严管、损害严惩、责任追究，实行资源有偿使用和生态补偿制度，完善考核评价制度，形成有利于生态文明建设的利益导向机制。

　　第八，建设幸福美好生活。要引导和鼓励县（市、区）发展产业集群，推进扩权强镇改革，提升县域经济发展的层次和水平。大力实施产业脱贫、就业脱贫、移民搬迁脱贫和保障脱贫，力争提前两年实现现行标准下贫困人口全部脱贫。实施更加积极的就业政策，健全收入分配调节机制，努力提高城乡居民收入。以扩大保障覆盖面、提高保障标准为重点，稳步提高社会保障水平。加强医疗卫生服务能力建设，广泛开展全民健身运动，统筹做好人口和计划生育工作。

　　第九，全面深化改革。要以经济体制改革为重点，努力在重点领域和关键环节改革上取得决定性成果，为经济社会发展提供持久动力和制度保障。健全宏观调节体系，完善财税管理体制，进一步转变政府职能。建立公平开放透明的市场规则，大力推进要素市场建设，加快形成统一开放、竞争有序的现代市场体系。积极发展混合所有制经济，促进多种所有制经济协调发展。

　　第十，全面依法治市。要坚持运用法治思维和法治方式推动发展，增强全民法治观念，深入推进依法行政，努力营造公平公正的法治环境，建设法治瓷都。推进平安瓷都建设，促进多层次、多领域依法治理，提高社会治理法治化水平。发展社会主义民主政治，最大限度凝聚全社会推进改革发展、维护社会和谐的共识和力量。

　　《纲要（草案）》还就加强党的领导、完善规划落实机制、形成规划实施合力提出了要求。

　　三、2016年工作安排

　　今年是全面建成小康社会决胜阶段的第一年，我们将深入贯彻省委十三届十二次全会、全省经济工作会议和市委十届十二次全会精神，紧紧围绕市委提出的发展定位，按照产业更强、城市更美、文化更特、生态更优、民生更实的发展要求，坚持国际化思维、全域化规划、项目化推进、景区化建设、一体化发展，精心实施“三大战役”深入推进年活动，努力实现“十三五”经济社会发展的良好开局。主要预期目标是：生产总值增长8.5%以上，财政总收入增长9%，规上工业增加值增长9%，固定资产投资增长15%，社会消费品零售总额增长11%，实际利用外资增长10%，外贸出口增长2%，城镇居民人均可支配收入增长9%，农村居民人均可支配收入增长10%，居民消费价格涨幅低于3%，单位生产总值能耗、主要污染物排放完成省政府下达目标，人口自然增长率低于9.7‰。

　　为实现上述目标，将主要采取以下举措：

　　（一）深入推进新型工业化

　　加快产业集聚。围绕做优汽车、做大航空、做强陶瓷产业的总体要求，坚持“产城融合、集约发展、合作共赢”，加大基础设施建设力度，完善配套服务功能，做优工业发展平台，全力推进高新区、陶瓷工业园、北汽昌河新基地和配套产业园建设，加快实施昌飞总装园、北京通航直升机、名坊园、邑山陶瓷综合体等重大产业项目，进一步完善产业配套，延伸产业链条，加快优势产业集群化步伐。完善运行调度和企业帮扶机制，帮助实体经济降低成本。大力扶持中小企业发展，力争两年内建成10个标准化创业园、发展20户“专精特新”中小企业。

　　实施创新驱动。加大科技创新投入，完善协同创新体系，充分发挥科技创新对产业转型升级的支撑作用。支持企业建立研发机构，帮助企业组建各类工程技术研究中心与重点实验室，培育一批创新型龙头企业、科技型中小企业，增强企业自主创新能力。探索建立科技成果转化产业基金，调动科技人员创新创业积极性，加快科技成果转移转化。积极推进“互联网＋”行动，推动物联网、大数据、云计算等在制造业领域的应用，加速工业化、信息化深度融合。加快企业技术升级改造，实施一批重大技改项目，支持汽车、航空、陶瓷等主导产业提高工艺、装备、能效水平。

　　坚持绿色发展。深入推进生态文明先行示范区建设，加快构建环境友好的绿色产业体系，走出一条绿色循环低碳发展的新路。大力发展节能环保产业，积极推广清洁生产及节能减排新技术、新装备，加快陶瓷、水泥、医药、化工等传统制造业技术改造。鼓励企业、园区推行循环生产方式，提高资源综合利用水平。严格按照国家产业政策，把好能耗和环保准入关。加强污染源综合治理，加大环保执法力度，加快生态红线划定工作。加强水资源管理、保护、开发，全面推进节水型社会建设。

　　（二）全面扩大对外开放

　　主动融入大开放格局。把握国家“一带一路”、长江经济带等战略机遇，创新开放模式，拓宽开放领域，提升开放程度。加快九景衢铁路建设，全面开工建设景德镇北站房和高铁商务区，加快机场迁建和昌景黄高铁前期工作步伐，新增景德镇至昆明、杭州航线，启动景南收费站及出入口连接线工程，不断提升对外通达能力。充分发挥陶瓷文化的独特优势和魅力，全面拓展与全球创意网络城市合作，提升“世界手工艺与民间艺术之都”影响力，并通过文化搭桥，推动各领域全方位开放。全面推进通关一体化，进一步提升外贸便利化水平。加大对外向型企业的帮扶力度，扶持医药化工及压缩机、陶瓷、茶叶等产品出口，着力培育成长型出口企业，鼓励有条件的企业走出去，开拓国际市场。

　　加大招商引资力度。大力开展产业招商。工业要重点围绕汽车、航空、陶瓷等产业上下游配套开展产业链招商，不断壮大主导产业集群；现代农业、服务业要重点引进龙头企业整合产业资源，提升产业档次和规模。积极实施精准招商。紧盯跨国公司、央企、知名民企投资动向，主动上门对接；积极参与全国、全省各类招商推介活动，并主动赴长三角、珠三角等地举办形式多样的专题招商推介会；充分利用驻外机构、商会等开展代理招商、以商招商，并实施“景商返景”计划。进一步改善投资环境，提高服务水平，加快项目落地开工。

　　增强筹资融资能力。在发挥国有资本引领作用的同时，按照“非禁即入”原则在产业投资、基础建设和一般公共服务领域推行PPP模式，拓宽项目融资渠道，促进政府主导直接融资向市场主导直接与间接融资相结合转变。加强对国家政策的研究，结合我市实际和发展需要，着力谋划并向上争取更多的项目和资金支持。

　　（三）着力提升城市品质

　　精心打造功能片区。昌南拓展区要启动北片区路网和西城区水系整体改造工程，建成市民服务中心、社区服务中心、学校、医院等公建项目，加快推进绿地陶瓷文化旅游城等项目建设，改造国际陶瓷创意中心。高铁商务区要加快主干道和站前广场建设，为2017年动车通车创造条件。赣东北物流园要加快基础设施建设和家居建材、农副产品批发、物流仓储等项目的引进落地。昌江、珠山两个区域副中心要加快前期工作步伐。统筹地上地下基础设施，开工建设2条地下综合管廊。

　　保护提升老城区。按照留住历史文脉、体现古镇特色的要求，继续推进老城区保护利用工程，联动推进沿江东路保护与彭家弄老街区综合利用项目。加快中心城区更新改造，重点对“老街区、老厂区、老窑址”、“城市出入口沿线、城区主干道沿线、滨江景观带沿线”以及城中村实施环境整治和整体提升，改造部分道路街区外立面。优化城市交通路网，加快推进景东大道二期、景德大道延伸工程，拓宽改造206国道丽阳至唐英大道段，实施一批城市道路绿化提升、断头路优化贯通、破损路面维修改造工程。加快推进观音阁至瓷都大桥段及吕蒙防洪堤、飞虹大桥及飞虹大道、洋湖水厂搬迁及配套管网工程。中心城区110千伏以下电线逐步下地。同时，完善一批事关民生和城市形象的市政基础设施，实现建筑形象美、景观环境优、基础设施全、户外广告靓。

　　提高城市治理水平。以“五城同创”推动城市治理方式转变和治理能力提升，着力增强城市宜居性。结合立法权限下放，加快城市管理法制化进程。强化规划引领作用，推进“多规合一”，提高规划覆盖率。启动老城区、迎宾路周边、高新区组团、高铁商务区等区域城市设计工作，塑造城市特色。积极推进“天网工程”建设，不断扩大视频监控覆盖面。充分发挥属地管理优势和“数字城管”平台作用，实行全覆盖管理，对珠山中路、珠山西路、莲社北路实行精细化管理，加快城市管理由“单一向互动、粗放向精细、突击向长效”转变。深入开展市容环境专项治理，对施工扬尘、建筑垃圾、渣土运输等进行源头管控，落实“门前三包”制度，集中整治夜宵摊点和占道经营，全面推进城区主次干道店招广告规范改造。深入开展交通拥堵治理和停车规范管理，积极做好全国文明城市测评和省级文明城市复查考评，争取测评成绩排位前移，力争成功创建国家森林城市。以开展违规自建、参与“两违”和插手干预打击“两违”工作专项治理为突破口，继续保持打击“两违”的高压态势，深入开展房地产市场专项整顿，规范小区物业管理。

　　（四）加快构筑“三农”发展新格局

　　夯实农业生产基础。继续推进我市农业三大体系及粮食高产创建项目建设。落实粮食安全责任制，加强耕地保护，全面完成永久性基本农田划定工作，整合资金继续建设高标准农田9.2万亩。推进浯溪口水利枢纽建设，确保年底竣工。大力开展农田水利基本建设，对新出险的37座小型病险水库进行除险加固，实施中小河流治理项目。粮食播种面积和总产量分别稳定在139万亩和12.5亿公斤以上，完成造林4万亩。

　　推动农业产业升级。充分发挥生态和资源优势，在蔬菜、茶叶、休闲农业等领域打造特色鲜明、链条较长、带动力强的优势农业产业集群。扩大农业经营主体规模，新增农民专业合作社106家，培育家庭农场72家。扩大农产品展示展销，提高农产品市场竞争力。在稳定蔬菜、茶叶产量的基础上，大力推进精深加工和品牌整合，提升产业层次和效益。加快发展休闲农业，争创1个国家级示范点、2个省级示范点。加快推进乐平国家级现代农业示范园区和浮梁、昌江2个省级示范园区建设，充分发挥园区对转变农业生产方式的引领带动作用。

　　建设和谐秀美乡村。继续抓好重点村、亮点村建设，着力提升新农村建设的品质。重点打造1个省级示范中心村、7个市级重点中心村，加快推进5个省级百强中心镇扩权强镇试点。大力开展农村人居环境整治，严把垃圾处理源头和终端两个环节，基本实现垃圾处理城乡一体化覆盖，有序开展农村生活污水处理工作，浮梁县、昌江区农村生活垃圾专项治理要通过省级验收。完成浯溪口水库移民搬迁安置。全力实施精准扶贫攻坚，提高扶贫对象自我发展能力。

　　（五）不断壮大现代服务业

　　努力做旺旅游产业。编制完成旅游名城发展系列规划，促进旅游与陶瓷、文化、商贸、农业等产业深度融合，推动旅游产业集群化发展，努力打造“国际知名、国内一流”的旅游目的地。大力发展城市旅游，创新发展文化旅游，提升发展乡村旅游，不断丰富旅游业态。新建和创评一批高档旅游饭店，打造一批农家乐联合体和示范旅游乡、村，开发一批体现瓷都特色的旅游商品。积极培育壮大旅游市场主体，加快筹建旅游集团，打造实力雄厚、竞争力强的区域旅游龙头企业。依托老街区、陶溪川、名坊园、浯溪口水库等载体打造旅游精品项目，力争引进具有轰动性、震撼力的大型旅游综合体项目。扩大旅游区域合作与营销，创新旅游推介形式，壮大旅游产业规模。

　　加快发展金融服务业。继续加大金融机构引进力度，完善金融服务体系，积极争取设立金融租赁公司、基金公司等新型金融机构。鼓励融资担保、小额贷款等金融中介机构加快发展，壮大业务规模。推进普惠金融发展，努力构建乐平市普惠金融示范区。完善政银企对接机制，继续扩大“财园信贷通”、“惠农信贷通”和实体经济还贷周转基金业务，鼓励银行业加大对中小微企业和“三农”信贷投放。加快宏柏化学、昌兴航空、开门子肥业等企业上市或“新三板”挂牌筹备步伐。继续优化融资平台建设，充分发挥航空、陶瓷等产业发展基金的杠杆作用和乘数效应，广泛吸引各类资本投入实体经济。

积极拓展服务业领域。坚持把文化作为对外竞争发展的软实力和转型升级的内动力，充分挖掘利用丰厚的陶瓷文化资源，更大程度地转化为经济优势。以陶溪川、名坊园、三宝瓷谷等集聚区为重点，打造文化创意产业集群。引进北辰集团组建国内一流的专业会展公司，并以此为契机转变瓷博会运作模式，推动会展经济进入快车道。加快发展电子商务，建立垂直电商交易平台和电商产业园区，扶持壮大一批电商企业，促进电商与陶瓷、现代农业、旅游等产业链的高效整合。建设陶瓷电商示范基地，承办好第二届中国陶瓷电商峰会。加快发展现代物流、工业设计、技术研发、融资租赁、信息服务等生产性服务业，助推产业向价值链高端提升。适应消费结构升级新形势，推动商贸、餐饮、娱乐、养老、家政等生活性服务业精细化、便利化、品质化发展，增加有效服务供给。

　　（六）统筹推进民生和社会事业

　　加大民生保障力度。真诚回应群众关切，增加民生资金投入，提高公共服务质量，切实增强群众获得感。重点为民办好10件实事：1．发放小额贷款4.3亿元，为2570个公益性岗位发放补贴。2．改造10条老城区道路和4条人行道。3．完成50座旱厕改造，新建7座站厕合一垃圾中转站和2座公厕。4．新建、改造5个街心花园。5．新建400套公租房，发放租赁补贴7300户，实施棚户区改造7000户以上。6．购置20辆新空调公交车，建设（改造）60座公交候车亭；新增昌南拓展区公交线路，加密高新区、陶瓷工业园公交线路；开通至婺源高铁站直通车。7．完成1.8万人农村饮水安全改造工程。8．建设10条全民健身路径、5个农民健身工程。9．建设20个城乡居家养老服务中心（站）。10．中心城区增设3个汽车加气站、建设一批汽车充电桩。

　　协调发展社会事业。优化城乡教育布局，实施义务教育阶段学校标准化建设、薄弱学校改造等工程，促进义务教育均衡发展。加强公办幼儿园、优质特色高中建设。创新校企合作模式，提高职业教育针对性。以申报世界文化遗产为龙头，做好御窑厂大遗址保护工作。加强文化遗产保护利用，完成国家级项目《景德镇手工制瓷技艺》数字化保护。深入实施文化惠民工程，积极推动基本公共文化服务标准化、均等化。推进公立医院改革，加强医疗服务体系建设和医疗质量管理，提升医疗卫生服务能力。稳妥有序实施两孩政策，推进出生人口性别比综合治理。深入开展全民健身活动，加快建设、改造体育场馆，加快省运会筹备工作。加快二轮《市志》编修，积极推进档案馆、地方志馆数字化建设。建立公共检测技术平台“大数据”信息安全机制。加强防灾减灾科普教育，增强群众防灾意识。

　　维护社会安定和谐。围绕保障社会公正、促进社会诚信、维护社会秩序三大重点，加快“法治景德镇”建设。深入开展法治宣传教育，加强普法工作，加快社会信用体系建设。加强社区规范化建设和网格化管理，推动社会建设和管理重心下移，提高社区服务水平。完善立体化社会治安防控体系，充实规范辅警队伍，严厉打击违法犯罪，切实增强公众安全感。改进信访和调解工作，完善依法维权和矛盾化解体系，健全利益表达、协商沟通、行政裁决和救济救助机制，努力实现风险预警和矛盾化解常态化。完善应急管理体制机制，增强应急处置能力。深化重点行业领域隐患排查治理和安全生产专项整治，加强食品药品和农产品质量安全监管，加强重大活动、节假日、人员密集场所等公共安全管理，坚决遏制重特大事故发生。支持驻景解放军和武警部队建设，做好国防建设、国防动员、人民防空和优抚安置等工作，大力推进军民融合发展。

　　（七）全面深化各项改革

　　坚持把改革创新作为推动发展升级的根本动力，今年要在落实已出台改革措施的同时，重点推进以下改革：优化行政审批流程，进一步缩短办结时限、提高办事效率。建好中介机构服务平台，打造中介服务超市。积极推进公共资源交易平台诚信机制建设，完善招标代理机构信誉评价机制。全面完成第一批行政及参公单位公务用车制度改革。全面实施县以下机关职务与职级并行制度。全面启动机关事业单位养老保险制度改革，建立与企业职工等城镇从业人员统一的基本养老保险制度。大力推进财政资金统筹使用，盘活沉淀资金，提高资金使用效益。进一步完善县、乡财政管理体制，合理划分县、乡财政收支，保障基层政权基本需要。进一步深化国资国企改革，通过兼并重组发展壮大一批国有企业集团，积极引进战略合作伙伴发展混合所有制经济。全面实施市属企业负责人薪酬制度改革。全面完成农村土地承包经营权确权登记颁证工作。以警务前移为核心深化警务改革，建立规范化、信息化现代警务机制。多渠道引入社会资本办医、办校，支持社会资本参与养护中心、康复医院、护理中心及学校建设。

　　（八）加强政府自身建设

　　新的形势和任务对政府自身建设提出了更高要求。各级各部门要进一步解放思想，把解放思想作为全面深化改革的先导，以思想的再解放，推动环境改善和创新创业。要进一步强化依法行政，深入推进法治政府建设，坚持用法治思维和法治方法履行政府职能、规范行政行为、推动改革发展。要进一步改进工作作风，勤政为民、善谋敢为、务实高效，不断提高工作效率和服务质量。要进一步加强廉政建设，认真落实“一岗双责”，把纪律挺在前面，持之以恒落实八项规定精神，着力解决群众身边的不正之风和腐败问题。

　　各位代表，千里之行，始于足下！“十三五”规划描绘了一幅美好的“效果图”，要把它变成真正的“实景图”，需要我们保持定力、埋头苦干、久久为功。让我们在省委、省政府和市委的坚强领导下，努力实现“十三五”发展的良好开局，为提质翻番、率先小康打下坚实基础，为复兴千年古镇、重塑世界瓷都、保护生态家园、建设旅游名城、打造一座与世界对话的城市作出新的更大贡献！
